# English Adjectives and Estonian Nouns: Looking for Agreement?

**DOI:** 10.3389/fpsyg.2021.735232

**Published:** 2021-10-14

**Authors:** Daria Bahtina, Helin Kask, Anna Verschik

**Affiliations:** ^1^Department of Linguistics, University of California, Los Angeles, Los Angeles, CA, United States; ^2^School of Humanities, Tallinn University, Tallinn, Estonia

**Keywords:** language contacts, bilingual constructions, grammaticality judgement, Estonian, English, multilingualism, morphosyntactic integration

## Abstract

This study investigated how speakers of Estonian as L1 with varying degree of proficiency in English judge grammaticality of bilingual constructions *English adjective* + *Estonian noun* from the point of view of adjective agreement. Estonian is rich in inflectional morphology, and adjectives agree with nouns in case and number. The empirical evidence from English-Estonian bilingual speech shows that agreement is not always the case even when an English adjective fits into Estonian declension system. It is hypothesized that the higher proficiency in/exposure to English is, the higher is the acceptability of bilingual adjective phrases, and (non-)agreement does not play a role. To test this, an experiment was designed where the test corpus of 108 sentences consisted of real and constructed examples, both in agreement and non-agreement condition. Real sentences came from fashion and beauty blogs and vlogs. The test was administered online and the participants were asked to rate adjective acceptability. The hypothesis was confirmed: increased proficiency in English, together with younger age, had a positive correlation with acceptability of all adjective types, independent of adjective (non-)agreement. Residence and birthplace had a small effect on acceptability of some adjective types. Whether sentences were real or constructed, had only a minor effect. Male participants tended to assess real sentences lower, probably because of the topics typical for female blogs. Monosyllabic consonant-ending adjectives were exceptional, as their assessment did not depend on any factor. All in all, the study demonstrated that grammaticality judgment among the native speakers of the same L1 differs because of different degrees of bilingualism, and structural factors, such as compatibility with Estonian declension system, are not decisive. Thus, it is not clear what an ideal native speaker is.

## Introduction

The interest toward how a non-native speaker differs from a native speaker and whether native(-like) competence can be achieved is explicitly stated in the context of SLA/bilingualism/multilingualism research with the focus on languages that are acquired later than L1 (i.e., L2 etc.). Since the 1980s the notion of a monolingual native speaker as an ideal and a yard stick has been questioned by some scholars (e.g., Skutnabb-Kangas, 1984; Rampton, 1990) because it is not always clear what “native” is: at times, the language acquired first is not one's dominant language, and internal identification may not coincide with external (that is, how other speakers assess a given speaker). Piller (2002) showed that so-called non-native speakers may pass for native speakers, as quite a lot depends on whether those who assess have a prior knowledge of the fact that the language in question was not L1 for the person whom they assess. In the same spirit, Gnevsheva (2017) noted that sometimes so-called non-native speakers may be perceived as speakers of different regional varieties.

Cook (2002) and Dewaele (2017) suggest the term “language user,” i.e., L2, L3 or, as Dewaele (2017) has it, LX user rather than learner because languages may be acquired in a variety of ways, also without explicit learning. The notion of multicompetence, introduced in the early 1990s (see more in Cook, 2016) was a useful contribution to the field because it shed light on the monolingual bias. First, a multilingual user is not a sum of several monolinguals, and a multilingual speaker is not an imperfect version of a monolingual speaker (Murahata et al., 2016; Dewaele, 2017). Second, multilinguals have several linguistic systems in their mind, which renders their cognition and perception different from those of monolinguals who have only one linguistic system. Therefore, the latter are unable to juxtapose and draw parallels between the systems. It implies, among other things, that focusing on a particular LX without considering all other languages of a given individual gives a rather patchy picture. Important as this may be, the debate on native competence is centered around languages acquired later than L1, such as SLA, language pedagogy, teachers who are or are not native speakers of the language they teach and so on.

Quite remarkably, contact linguistics was not a part of the debate, probably because its scope and purpose are different from that of SLA and, at times, of bilingualism/multilingualism research. First, contact linguistics is not concerned with ways to achieve target acquisition; by definition, the discipline is concerned with contact-induced language change. If non-target acquisition occurs, in contact linguistics this is not relevant for comparison with an ideal native speaker; the focus is rather on the fate of this new variety (becoming an ethnolect/in-group register, diffusion into the mainstream, and so on). Multilingual communities have their own norms and what looks like imperfect acquisition from a synchronic point of view may have significant effects on the mainstream variety in a diachronic/historical perspective (“incomplete” acquisition of Baltic by speakers of Finnic that yielded Latvian is a textbook example, see Thomason and Kaufman (1988, p. 239) and references therein). In other words, the mistakes of today are the grammar of tomorrow.

Second, contact linguistic research often puts centerstage changes in L1, while SLA deals with L2. Thus, the other side of the coin is the change of perception of L1 among so-called native speakers with some proficiency in L2 (LX). For instance, research on Netherlands Turkish (Dogruöz and Backus, 2009) has demonstrated that some contact-induced features that have emerged under the impact of Dutch are a new norm, while for speakers of Turkish in Turkey these features appear erroneous and, therefore, non-native.

This discrepancy between the contact linguistics view on language change, including L1, and the notion of a monolingual native speaker, still common in SLA research, is the starting point for this article. The focus is on perception of adjective-noun (non-)agreement in bilingual phrases English adjective + Estonian noun, for instance *fancy-d kinga-d* (fancy-PL.NOM shoe-PL.NOM) “fancy shoes.” Estonian is agglutinating-fusional language with highly developed inflectional morphology, where adjectives agree with nouns in case and number. From a structural point of view, English adjectives that fit into Estonian declension system would take on Estonian inflections. However, it is not always the case (Kask, 2019). We assume factors other than structural compatibility play a role here.

A number of approaches to multilingual language use focus on formal distinctions and classify phenomena accordingly (i.e., lack of integration = code-switching and integration = borrowing). Albeit we do not agree with the formal constraints proposed in the Matrix Language Frame model (Myers-Scotton, 1997), we believe that the description of the following options is empirically right: a stem from Embedded Language may retain inflections from that language, it can take on inflections from the Matrix Language (i.e., the main language of the clause), and it can remain without any markers (so-called bare forms). A code switched item can become a borrowing, if it is useful and if it gains currency and becomes a new norm. Neither is this project concerned with conventionalization (and codification) in monolingual use, as customary in Anglicisms research. It will be discussed below that multilingual speakers do not necessarily need the same mechanisms of integration as monolinguals do. In terms of classification, we can use Muysken's (2000) typology, in which English adjectives can be treated as insertional code-switching, as opposed to alternational code switching and congruent lexicalization. We concur with usage-based approaches to contact-induced language change (Zenner et al., 2019) in general and to borrowing in particular (Backus, 2012, 2014). In these approaches, the difference between one-word code-switching and borrowing cannot be described in formal terms. Instead, it is rather a matter of frequency and conventionalization than any formal criteria. This is why our underlying research question is centered around the acceptability of various English adjectives as a harbinger of new norms in the making.

Estonia is a small country with a population of 1.3 million, of which speakers of Estonian as L1 make up roughly 68%. Multilingualism is not new in Estonia, German and Russian have been sociolinguistically dominating languages for centuries (Ariste, 1981). Language contacts between Estonian and English are relatively recent, starting from the 1990s after the restoration of independence, and the changes are on-going. Recent decades have witnessed growing competence in English among younger Estonian-speakers: 84% of Estonians in the age group 15–24 and 64% in the age group of 25–39 claimed their ability to speak English (Kruusvall, 2015). According to the Housing and Population Census 2011, 38.3% of Estonian residents have indicated their knowledge of English, and 20% claim they do not speak English. However, research has shown that the percentage of Estonians who are not proficient in English is decreasing and the percentage of Estonians who have active knowledge of English is rising (Koreinik and Tender, 2013, p. 86; Kruusvall, 2015, p. 78). Students consider English as the most important subject at school (Tammemägi and Ehala, 2012, p. 249), and 96.5% of Estonian students in upper secondary general education learnt English as a foreign language (Eurostat, 2017). In addition, English is quite often also acquired informally *via* the internet and popular culture. The differences in linguistic resources between generations in Estonia is quite obvious. Thus, it may be assumed that Estonians who do not use English and those who use English on a regular basis and produce multilingual speech might have different kinds of linguistic awareness.

We employ the usage-based approach to contact-induced language change, in particular, cognitive contact linguistics (Backus, 2014; Zenner et al., 2019), assuming that there is no dichotomy between competence and performance, and competence is shaped by usage. In such approaches, language use and cognition are interconnected: new circumstances affect language use that in its turn affects cognition and perception of norms. In other words, grammar is neither pre-programmed nor static but is shaped by usage. This is in line with the above-mentioned idea that a multigual's knowledge of their L1 differs from that of a monolingual because input, output, linguistic resources, communicative patterns etc. are different. Taking this into consideration, we assume that those who use English more would be less affected by (in)compatibility of English adjectives with the Estonian declension system and would accept bilingual adjective phrases regardless of the shape of adjectives.

In order to check our assumption, we designed an experiment where we tested perception of real and constructed sentences containing English Adj + Estonian N, both in the agreement and the non-agreement conditions, among various speakers of Estonian as L1 (see Data and Method section). One of the reasons why we opted for an experimental study is an almost complete lack of Estonian-English bilingual speech corpora (our own corpus of bilingual blogs and vlogs is respectively 275,000 and 48,000 words)^1^. By bilingual blogs and vlogs we mean that all entries contain either overt elements from English (insertional or alternational code-switching) or loan translations, constructions, patterns etc. More on Estonian-English blogs and vlogs see in Kask (2019, 2021), Verschik and Kask (2019). Personal blogs and vlogs are not restricted with the notion of “correctness” and, therefore, give a picture of naturalistic language use.

Our research questions are as follows:

(1) Whether there is a difference in grammaticality judgement depending on proficiency in and exposure to English;
(2) whether there is a difference in perception of real and constructed examples;
(3) whether there is a difference in perception of the group of English adjectives that fit into Estonian declension system but do not have Estonian inflections in our corpus.

The article is organized as follows. In Adjectives in bilingual phrase *English Adj* + *Estonian N* section, we describe adjective declension types in Estonian and the findings of the only existing empirical study by Kask (2019). Data and Method section focuses on the experiment methodology. The results are presented in Results section, followed with discussion and conclusions in Discussion section.

## Adjectives in bilingual phrase *English Adj + Estonian N*

English and Estonian are typologically different: English is an isolating analytic language, while Estonian is an agglutinative language with fusional tendencies (Erelt, 2007, p. 7). Estonian has a rich inflectional morphology with 14 grammatical cases. In Estonian adjectives agree with nouns both in number and case, for instance: s*uur-te-sse maja-de-sse* “into big houses” (big-PL-ILL house-PL-ILL) where both the adjective and the noun are in the plural illative. In four cases such as terminative, essive, abessive and comitative, the agreement is in number only, the adjective is present as a stem (technically, the genitive stem, from which all oblique cases, except the partitive, are formed): *suure maja-ga* big.GEN house-COM “with a/the big house,” *suur-te maja-de-ga* big-PL.GEN house-PL-COM “with (the) big house.” However, English lacks grammatical cases altogether, and adjectives do not agree with nouns in number: *into big house-s* (big.SG house-PL) the noun is in the plural but the adjective remains in the singular.

Based on that, it would be expected that in a bilingual phrase English adjectives would agree with Estonian nouns, at least in the event when an English adjective is compatible with Estonian adjective declensions. A study by Kask (2019) showed that in bilingual blogs and vlogs the English adjective does not agree with the noun if it does not fit into the Estonian declension system. There is a tendency for agreement, if English adjectives are phonotactically similar to Estonian adjectives and, therefore, are structurally compatible with the declension system. Yet there are instances where the English adjective is compatible with the Estonian declension system but, contrary to expectations, does not receive Estonian inflections.

In the empirical data discussed in Kask (2019), seven types of English adjectives emerged, the types are presented in Table 1. Out of these, five types fit into Estonian declension system, so it would be expected that these adjectives agree with the Estonian noun. However, types marked with asterisk showed variation and there were several instances of the adjective being not integrated. Consider Examples (1) and (2) with the adjective *basic* used by two vloggers (Kask, 2019, p. 93), where *basic* can be declined as *lapik* “flat”.


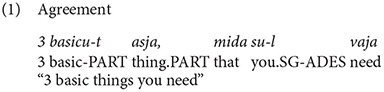



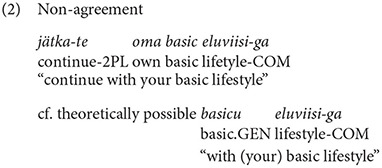


**Table 1:** Types of English adjectives presented with Estonian prototypes and sentence examples.

**Type**	**English example**	**Estonian prototype**	**Sentence example**
**(A) Types that fit into the Estonian declension system and agree with the noun**			
Monosyllabic, consonant ending	*deep*	*hell* “tender”	*Olin juba oma peas järgmist **diipi blogipostitus-t*** (deep.PART blogpost-PART) *kirjutamas*.
			“I was already writing the next deep blogpost in my head”
Disyllabic, ending with [i]	*fancy*	*tubli* “diligent”	*Imetlesime **fancy-sid tänava-i-d*** (fancy-PART.PL street-PART-PL).
			“We were admiring fancy streets.”
Disyllabic in nominative, ending with [k]*	*basic*	*lapik* “flat”	*Need on üsna **basicu-d teksa-d*** (basic-NOM.PL jeans-NOM.PL)
			“These are pretty basic jeans”
Monosyllabic, ending with obstruent, subject to stem alternation*	*flat*	*pikk* “tall”	***Flati-d rehvi-d*** (flat-NOM.PL tire-NOM.PL) *mind eriti kaugele ei sõiduta*.
			“Flat tires won't take me very far.”
Minimally disyllabic, ending with consonant both in writing and in pronunciation*	*random*	*ilus* “pretty”	*Mul on väga **randomi-d ehte-d*** (random-NOM.PL jewlery-NOM.PL) *kõrvades hetkel*.
			“I'm wearing very random jewelry in my ears right now.”
**(B) Types that fit into Estonian declension system but do not agree with the noun**			
Ending with vowel in spelling but with consonant in pronunciation	*beige*	*kõrb* “dun”	*Loosin välja ühe **beige nokatsi*** (beige cap.GEN).
			“I will give away a beige cap.”
Ending with [v] in pronunciation and are therefore similar to Estonian present participles	*impressive*	*hariv* “educational”	*Siinkohal tahaks teha sellise **appreciative momendi*** (appreciative moment.GEN).
			“Here I would like to have an appreciative moment”

Panel (A) shows adjectives that agree with nouns and panel (B) shows adjectives that do not; adjective types marked with an asterisk (*) show variation.

In Example (1), the adjective *basic* receives Estonian partitive marker, while in Example (2) it remains unmarked.

Test sentences in this experiment were constructed based on the types described in Table 1, which presents types of English adjectives as far as their compatibility with the Estonian declension system is concerned. Panel (A) showcases adjective types that fit and agree with Estonian nouns, panel (B) shows types that do not. Some types are in principle compatible, yet according to the empirical evidence they either exhibit variation or do not agree with nouns (Kask, 2019, p. 102, 106–115), these adjective types are marked with an asterisk (*).

## Data And Method

Everyone who considered themselves an L1 speaker of Estonian qualified as a respondent. It turned out that all respondents had at least some proficiency in English. The condition was mentioned in the introductory part of the questionnaire, and 568 persons responded. In the introductory part it was emphasized that there is no right or wrong answer, and the respondents should just choose the answer that they consider appropriate. Participants who skipped the first six background questions were discarded from the analysis, which led to 401 respondents considered in this paper. An overview of the sociodemographic information can be found in Supplementary Appendix A (general composition) and Supplementary Appendix B (English proficiency); other relevant info is presented in Sociodemographic Basics section.

The questionnaire was created *via* SurveyMonkey and was accessible online in the period from February 19 to March 8, 2021. The link was distributed *via* Tallinn University School of Humanities Facebook page as well as by the authors through their social networks. The background information for each participant was collected *via* questions about age, gender, and place of birth and residence; the latter were categorized into major cities, Tallinn and Tartu, smaller urban areas, rural areas, and abroad. The survey contained a number of questions targeting self-reported proficiency in English (comprehension and production), active language use (speaking and writing), and more passive exposure (listening and reading). We provide an English translation of the survey questions and answer options in Supplemenatry Appendix C. Individual scores on these parameters had a significant correlation and were therefore normalized to be used jointly. We refer to this multicompetence factor as *proficiency in and exposure to English* or *English* for brevity.

The set of sentences given for assessment was composed in the following way. For every type described in Table 1 we selected a real sentence from the corpus described in Kask (2019) and constructed a counterpart: if the adjective in the real sentence agreed with the noun, we presented the same sentence with a non-agreeing adjective, and vice versa. Since our aim was to have multiple test sentences for every adjective type, we constructed additional sentences in both agreement and non-agreement versions (5–10 sentences per type), arriving at a total of 108 sentences presented to the respondents. There were no time constraints for experiment completion: participants used a self-paced method to respond to each stimulus. All sentences were randomized during the experiment to eliminate any possible methodological biases.

Table 2 demonstrates how the set of test sentences were composed with a reference to the example of monosyllabic consonant-ending adjectives (Estonian prototype *hell* “tender”). Real examples (R) are preceded by an asterisk (*). All other sentences are constructed (C). For the sake of comparison, another set of test sentences is provided in Supplementary Appendix D.

**Table 2:** Test sentence examples for monosyllabic consonant-ending adjectives in the agreement (A) and the non-agreement (N) conditions.

**Type**	**Agreement (A)**	**Non-agreement (N)**
Monosyllabic adjectives ending in consonant (MS_C), Estonian prototype *hell* “tender”	* Lindexis olid coolid päiksekad müügil.	*Lindexis olid cool päiksekad müügil*.
	cooli-d päikseka-d	cool päikseka-d
	cool-PL.NOM sunglass-PL.NOM	cool sunglass-PL.NOM
	“there were cool sunglasses on sale at Lindex”	
	*Selle pintsliga saab ilusa cleani tulemuse*.	*Selle pintsliga saab ilusa clean tulemuse*.
	ilusa cleani tulemuse	
	nice.GEN clean.GEN result.GEN	ilusa clean tulemuse
	“with this brush you get a nice clean result”	nice.GEN clean result.GEN
	*Oma chilli olekuga jäi ta kohe kõigile meelde chilli olekuga*.	*Oma chill olekuga jäi ta kohe kõigile meelde chilli olekuga*.
	chilli oleku-ga	chill oleku-ga
	chill.GEN appearance-COM	chill appearance-COM
	“with his/her chill appearance s/he was remembered by everyone”	
	*Müün netioksjonil oma coole riideid*.	*Müün netioksjonil oma cool riideid*.
	cool-e riide-i-d	cool riide-i-d
	cool-PART.PL clothes-PL-PART	cool clothes-PL-PART
	“I am selling my cool clothes at a web auction”	
	*Filmin täna vlogi ühel väga funil teemal*.	*Filmin täna vlogi ühel väga funil teemal*.
	funi-l teema-l	fun teema-l
	fun-ADES topic-ADES	fun topic-ADES
	“Today I am shooting a vlog on a very fun topic”	
	*Freshid joogid on pärast trenni nagu rusikas silmaauku*.	*Fresh joogid on pärast trenni nagu rusikas silmaauku*.
	freshi-d joogi-d	fresh joogi-d
	fresh-NOM.PL drink-NOM.PL	fresh drink-NOM.PL
	“fresh drinks after a workout is just what the doctor ordered”	
	*Sel hooajal on moes warmides toonides kudumid*.	*Sel hooajal on moes warm toonides kudumid*
	warmi-de-s tooni-de-s	warm tooni-de-s
	warm-PL-INES tone-PL-INES	warm tone-PL-INES
	“this season knitwear in warm tones is in vogue”	

Real examples (R) are preceded by an asterisk (*).

The reason for testing both real and constructed sentences is the need to have some point of comparison. If all sentences were constructed, our results would speak only of metalinguistic awareness and would not cover instances where certain utterances were attested in real usage but mostly rejected by the respondents. This method was used by Verschik (2006) where references to other experimental studies in contact linguistics can be found.

The sentences were coded according to categories they represent: adjectives were divided into agreement vs. non-agreement categories and sentences were divided into real vs. constructed. Each sentence code also contained information about the more specific adjective type. For example, *MS_C_A_R* means that the adjective in the sentence belongs to the type “monosyllabic, consonant-ending,” the adjective agrees with the noun and the sentence is real. Acceptability judgements were collected using the Likert scales: respondents were asked to rate sentences on a scale from 0 (“nobody talks like this”) to 4 (“I would say this myself”). We present results as an aggregate based on each sentence category. The descriptive and inferential analyses were conducted using the open source statistical environment R (R Core Team, 2020), RStudio Version 1.4.1106, and the package lme4 (Bates et al., 2012).

## Results

### Sociodemographic Basics

Given the fact that the participants in our study were recruited without prior screening, we ran a series of preparatory tests to make sure that certain correlations between participant characteristics do not lead to erroneous interpretations.

First, we looked at the correlation between exposure to and experience in English vs. age. These two factors overlapped to a high extent: the younger the participant, the more English they seemed to have. Yet, when A Kendall's tau-b correlation was run to compare the two factors—age and English—only a moderate correlation was established (τ_b_ = 0.26, *p* < 0.01), which verified that the two factors can be used interchangeably only in about a quarter of the data, which means that age and English still affected the outcome separately. In other words, even if the most common participant profile is that of a young and fluent person, these two factors should not be collated for the purposes of data analysis.

We then investigated the relation between the experience of living abroad and English: the longer a participant spent abroad, the more likely they were to have a higher score in overall English, *F*_(1, 396)_ = 39.84, *p* = 0. Yet, living abroad cannot be treated as a precondition for an advanced English score in our dataset as there were participants with the highest score in English and no experience of living abroad.

There were several significant associations between acceptability rates and some of the sociodemographic characteristics that were true only for a subset of test sentences: residency, gender, and marginal associations for birthplace. We present the results of these factors in this section; other factors—age and English—showed more consistency within categories and adjective types and will be presented in Real vs. Constructed Sentences, Category and Acceptability Rates, Adjective Type and Acceptability Rates, Multivariate Analysis with Mixed Effects Modeling and What Can(not) be Explained by Sociodemographics and Structural Compatibility sections.

Residence played a role in acceptability rates of real sentences and some adjective types. A series of *post-hoc* Tukey's HSD tests revealed that in most cases it was Tallinn residents who had significantly higher acceptability rates than residents in the rural areas. There was a significant difference by residence in the real sentences, *F*_(4, 394)_ = 2.89, *p* = 0.02. According to the *post-hoc* test, Tallinn residents provided significantly higher acceptability rates for real sentences than the rural residents, *p* = 0.02, 95% C.I. = 0.04, 0.71. A significant difference between residency categories was also observed in the non-agreeing disyllabic adjectives ending with a consonant (*basic*), *F*_(4, 394)_ = 2.89, *p* < 0.01, and the non-agreeing disyllabic adjectives ending with an [i] (*fancy*), *F*_(4, 394)_ = 3.44, *p* < 0.01. The *post-hoc* test showed that the residents of Tallinn had a higher acceptability of non-agreeing disyllabic adjectives ending with a consonant (*basic*) when compared to the residents of rural areas, *p* < 0.01, 95% C.I. = 0.11, 0.72, and those currently residing abroad, *p* = 0.03, 95% C.I. = 0.02, 0.83. For the disyllabic adjectives ending with [i] (*fancy*), Tallinn residents had significantly higher acceptability rates than rural residents, *p* < 0.01, 95% C.I. = 0.09, 0.74. Finally, there was a significant difference by residency in the agreeing monosyllabic adjectives ending with consonant (*deep*), *F*_(4, 394)_ = 3.30, *p* = 0.03. The pattern was the same, Tallinn residents had significantly higher rates than rural residents, *p* < 0.01, 95% C.I. = 0.08, 0.63.

The birthplace of respondents also had a relationship with acceptability rates of some adjective types. The disyllabic adjectives ending with [i] (*fancy*) in the agreement condition demonstrated a difference across birthplace categories, *F*_(4, 394)_ = 2.70, *p* = 0.03. The other adjective type that had a significant difference by birthplace was the agreeing monosyllabic type ending with consonant (*deep*), *F*_(4, 394)_ = 3.76, *p* < 0.01. Respondents born in Tallinn rated this adjective type higher than respondents born in smaller Estonian towns, *p* = 0.02, 95% C.I. = 0.03, 0.61. Respondents born in Tartu also rated this adjective type higher than respondents born in smaller Estonian towns, *p* = 0.03, 95% C.I. = 0.03, 0.70.

Gender had a significant relationship with the acceptability rates of real sentences (as collected from the actual blogs and vlogs) but no other sentence category (i.e., constructed or agreement and non-agreement). Real sentences received lower acceptability rates from participants who identified as males (M = 1.55, SD = 0.86) than females (M = 1.83, SD = 0.82), *t*_(395)_ = 2.36, *p* = 0.02, *d* = 0.33; this effect was there regardless of age.

Gender also affected acceptability of the monosyllabic obstruent ending adjectives: female-identifying participants had higher acceptability rates than male-identifying participants for this adjective type both in the agreement and non-agreement conditions, *t*_(395)_ = 2.22, *p* = 0.03, *d* = 0.30 and *t*_(395)_ = 3.343, *p* < 0.01, *d* = 0.48. In the agreement condition, their mean scores were M = 1.88, SD = 0.83 for females and M = 1.61, SD = 0.91 for males; in the non-agreement condition, the means were M = 1.97, SD = 0.88 and M = 1.55, SD = 0.76, accordingly.

### Real vs. Constructed Sentences

**Figure 1:**
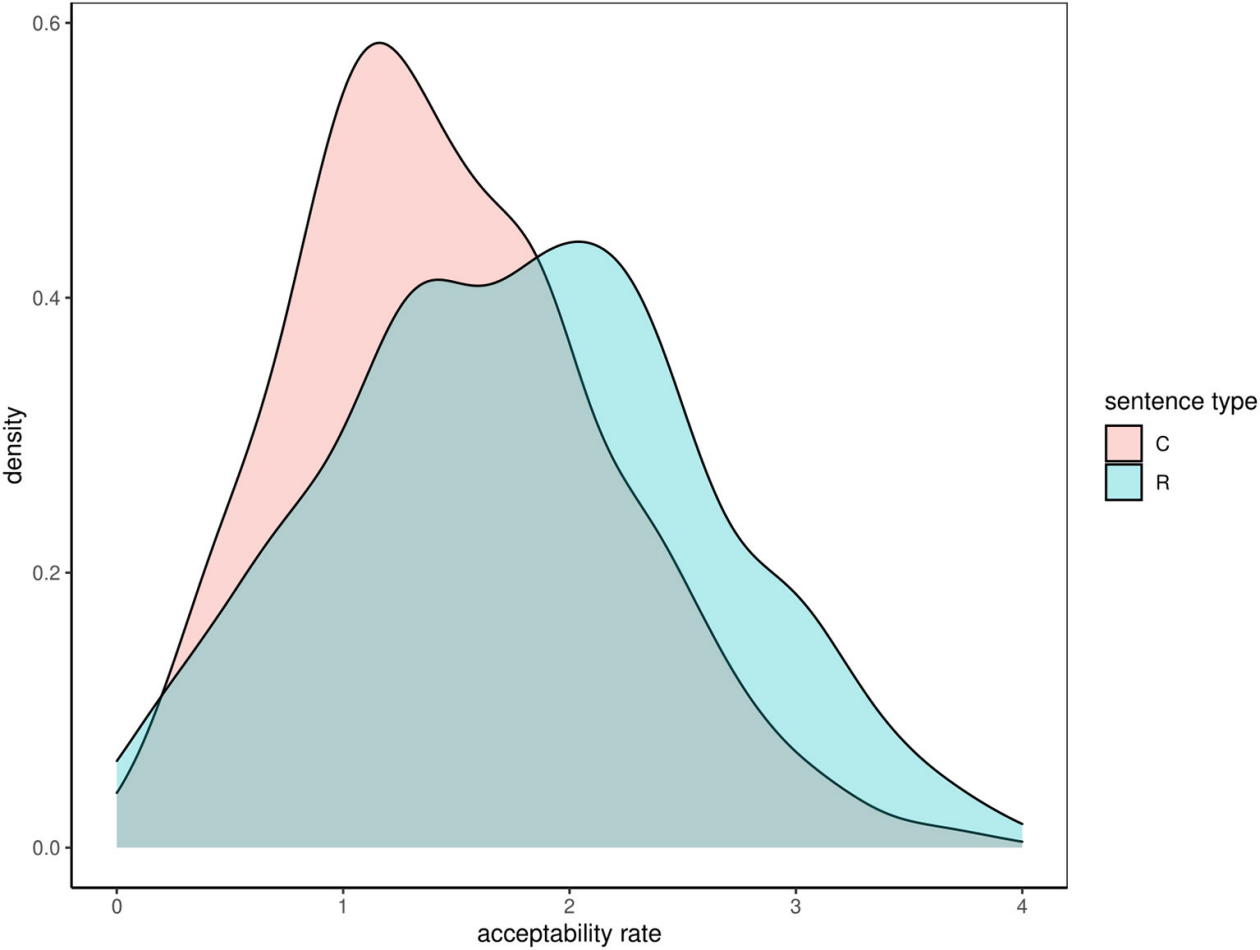
Difference between acceptability rates for real (R) and constructed (C) sentences.

The real sentences received somewhat higher acceptability ratings (M = 1.78, SD = 0.83) than the constructed sentences (M = 1.47, SD = 0.69). A Welch two-samples *t*-test showed that the difference was statistically significant but the effect size was rather moderate, *t*_(774.21)_ = 5.74, *p* < 0.01, *d* = 0.40. This difference is visualized in Figure 1.

### Category and Acceptability Rates

When analyzing the data by category (i.e., all adjectives, real and constructed sentences as well as sentences containing agreeing and non-agreeing adjectives), there was a strong correlation regardless of the category: the combined measure for English was positively correlated with acceptability rates and participants' age was negatively correlated with acceptability rates. These results are presented in Table 3.

**Table 3:** Regression results between acceptability rates across categories and two factors: proficiency in/exposure to English and age.

**Category**	**All adjectives**	**Real sentences**	**Constructed sentences**	**Agreeing adjectives**	**Non-agreeing adjectives**
English	0.23^***^	0.38^***^	0.21^**^	0.24^***^	0.22^***^
	(0.05)	(0.06)	(0.05)	(0.05)	(0.05)
	No. of observations		400		
Age	−0.22^***^	−0.32^***^	−0.20^***^	−0.23^**^	−0.20^***^
	(0.03)	(0.03)	(0.03)	(0.03)	(0.03)
	No. of observations		399		

*The table shows coefficients and significance codes (0*

“***” *0.001*

“**” 0.01 “*”, 0.1 “”). Standard deviations are reported in parentheses.

In other words, the higher the proficiency in and exposure to English, the more likely a participant to give a higher rate to adjectives in this data set. This was true for all categories of adjectives, whether the sentence was real or constructed or if the adjective agreed with the noun or not. The relationship was the opposite for age, meaning that older speakers systematically had a lower acceptability rate whereas younger speakers demonstrated a reliably higher acceptability rate.

### Adjective Type and Acceptability Rates

We also examined acceptability rates for separate adjective types and the correlation was largely the same: most adjective types received higher rates from respondents that were younger and more proficient in and exposed to English.

As shown in Table 4, all adjective types except one followed that pattern. The only exception was found for adjectives ending in a monosyllabic consonant (e.g., *deep*) when it was not in agreement with the noun. Neither age nor English as factors were able to account for varying acceptability rates of this specific adjective type. We discuss this adjective type in more detail in What Can(not) be Explained by Sociodemographics and Structural Compatibility section.

**Table 4:** Regression results between acceptability rates across adjective types and two factors: proficiency in/exposure to English and age.

	**DS_C_A**	**DS_C_N**	**DS_i_A**	**DS_i_N**	**DS_k_A**	**DS_k_N**	**MS_Obs_A**
English	0.22^***^	0.39^***^	0.38^***^	0.16^*^	0.27^***^	0.30^***^	0.20^**^
	(0.06)	(0.06)	(0.07)	(0.07)	(0.07)	(0.07)	(0.07)
	No. of observations			400			
Age	−0.21^***^	−0.32^***^	−0.31^***^	−0.13^***^	−0.28^***^	−0.29^***^	−0.19^***^
	(0.03)	(0.03)	(0.04)	(0.03)	(0.03)	(0.03)	(0.03)
	No. of observations			399			
	**MS_Obs_N**	**MS_C_A**	**MS_C_N**	**V_C_A**	**V_C_N**	**V_A**	**V_N**
English	0.26^***^	0.22^***^	0.00	0.18^**^	0.15^*^	0.20^***^	0.28^***^
	(0.07)	(0.06)	(0.07)	(0.06)	(0.06)	(0.05)	(0.06)
	No. of observations			400			
Age	−0.23^***^	−0.19^***^	−0.02	−0.20^***^	−0.17^***^	−0.22^***^	−0.25^***^
	(0.03)	(0.03)	(0.03)	(0.03)	(0.03)	(0.03)	(0.03)
	No. of observations			399			

*The table shows coefficients and significance codes (0*

“***”, *0.001*

“**”, *0.01*

“*”, *0.1 “ ”). Standard deviations are reported in parentheses*.

### Multivariate Analysis With Mixed Effects Modeling

Bivariate analyses show the association of acceptability rates with, on the one hand, respondents' age and, on the other hand, respondents' proficiency in and exposure to English. Our multivariate analysis uses lme4 package (Bates et al., 2012). This statistic assesses the ability of multiple independent variables (age and English) to predict—positively or negatively—a scalar dependent variable (acceptability rates of various categories of adjectives), while controlling for possible confounding effects, namely current residence and gender. Given the relatively small corpus sample (401 respondent), the model would not converge with more random effects, and following our usage-based approach, we selected current residence over birthplace. Our goal was to verify whether the main factors identified with the help of bivariate tests are still valid when random effects are included in the analysis.

Table 5 summarizes the main results of this linear mixed-effect model, testing the rate of acceptability outcome as predicted by age and English. The results show that both age and English are statistically significant predictors of the outcome, with opposite effects. Belonging to an older age band slightly decreases acceptability rates while English is positively associated with acceptability outcome.

**Table 5:** Linear mixed-effects model between acceptability rates across adjective types with two fixed effects (age and English in interaction) and two random effects (residence and gender).

**Category**	**All adjectives**	**Real sentences**	**Constructed sentences**	**Agreeing adjectives**	**Non-agreeing adjectives**
English~age	English^*^	English^*^	English^*^	English^*^	English^*^
	EST = 0.402, *SE* = 0.156	EST = 0.373, *SE* = 0.179	EST = 0.365, *SE* = 0.156	EST = 0.407, *SE* = 0.158	EST = 0.396, *SE* = 0.163
	English~age^*^		English~age	English~age^*^	English~age^*^
	EST = −0.084, *SE* = 0.038		EST = −0.084, *SE* = 0.038	EST = −0.084, *SE* = 0.039	EST = −0.082, *SE* = 0.040
Residence	VAR = 0.005, *SD* = 0.070	VAR = 0.007, *SD* = 0.085	VAR = 0.004, *SD* = 0.067	VAR = 0.004, *SD* = 0.068	VAR = 0.005, *SD* = 0.072
Gender	VAR = 0.005, *SD* = 0.070	VAR = 0.055, *SD* = 0.233	VAR = 0.004, *SD* = 0.065	VAR = 0.006, *SD* = 0.078	VAR = 0.007, *SD* = 0.085

The table shows significance codes for each statistically significant predictor (0 “***”, 0.001 “**”, 0.01

“*”, *0.1 “ “), estimates (EST) and standard errors (SE). We also report variance (VAR) and standard deviation (SD) for random effects*.

We did not expect the model to work for smaller subcategories due to lower counts but some specific sentence types showed a similar pattern: increased score in English was a predictor of higher acceptability rates, belonging to an older age band predicted the opposite. More specifically, age was a significant predictor for DS_C_N, English for DS_i_A and DS_k_N, and all three (English, age and their intercept) strongly predicted the outcomes for V_A and V_N adjective types. The following types did not show any significant relation with age and English when residence and gender were taken into account: V_C_N, MS_Obs_A, MS_Obs_N, as well as MS_C_A that did not reveal any patterns in the bivariate tests either. Other adjective types did not have sufficient variance to be included in the model (i.e., singularity warning). It should be noted that while these results were statistically insignificant or omitted due to singularity warning, the directionality was always the same.

**Figure 2:**
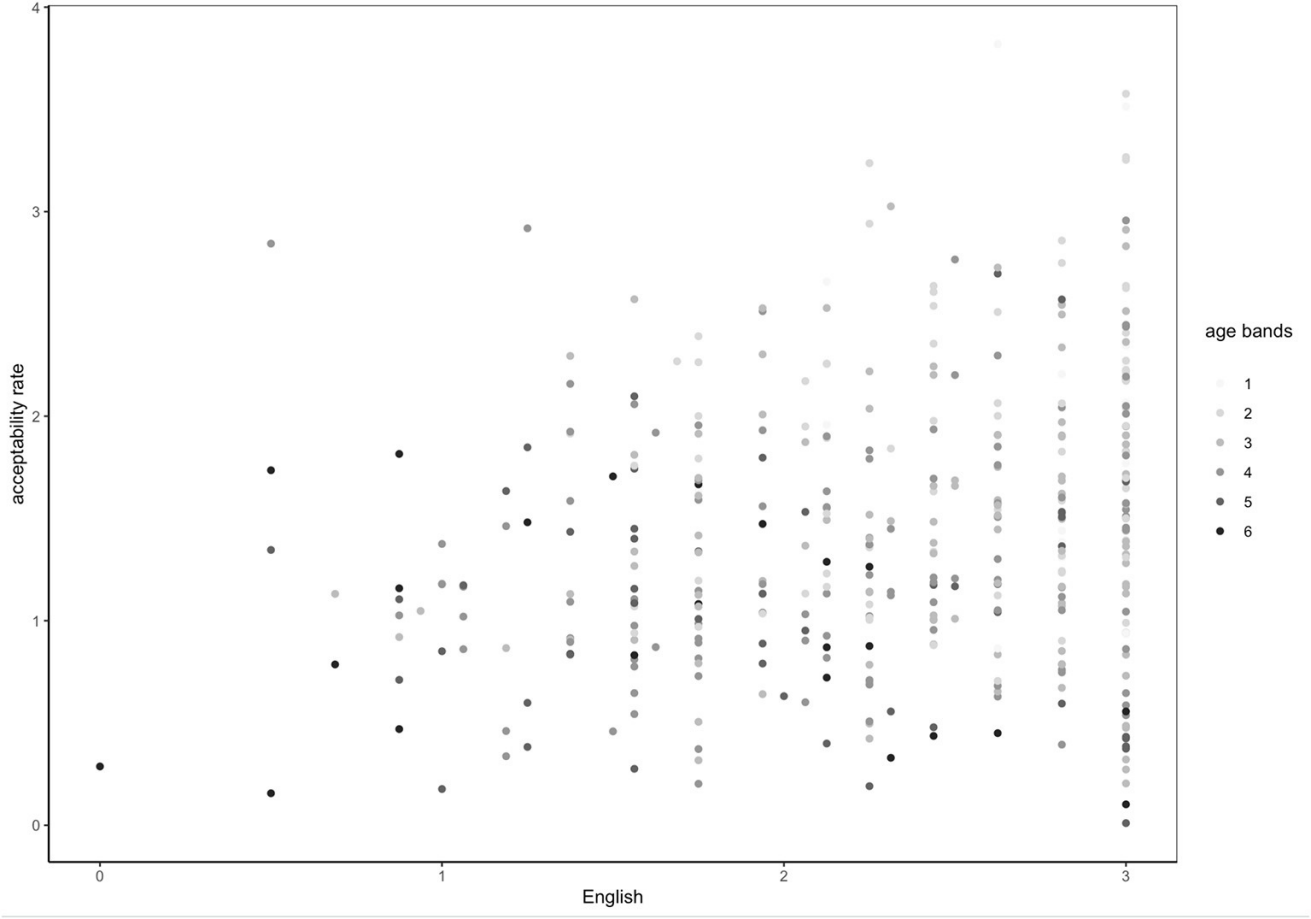
Acceptability rate of all adjectives by exposure to and proficiency in English by age bands (1 = under 20, 2 = 21–30, 3 = 31–40, 4 = 41–50, 5 = 51–60, 6 = 61–…).

Overall, multivariate analysis corroborates findings from bivariate tests in the following way: other factors being equal (i.e., residence and gender), age has a slightly negative correlation with acceptability rates of all adjectives while English is positively correlated. These results are visually represented in Figure 2: acceptability rates increase with English and slightly decrease with age even when English is high.

### What Can(not) Be Explained by Sociodemographics and Structural Compatibility

**Figure 3:**
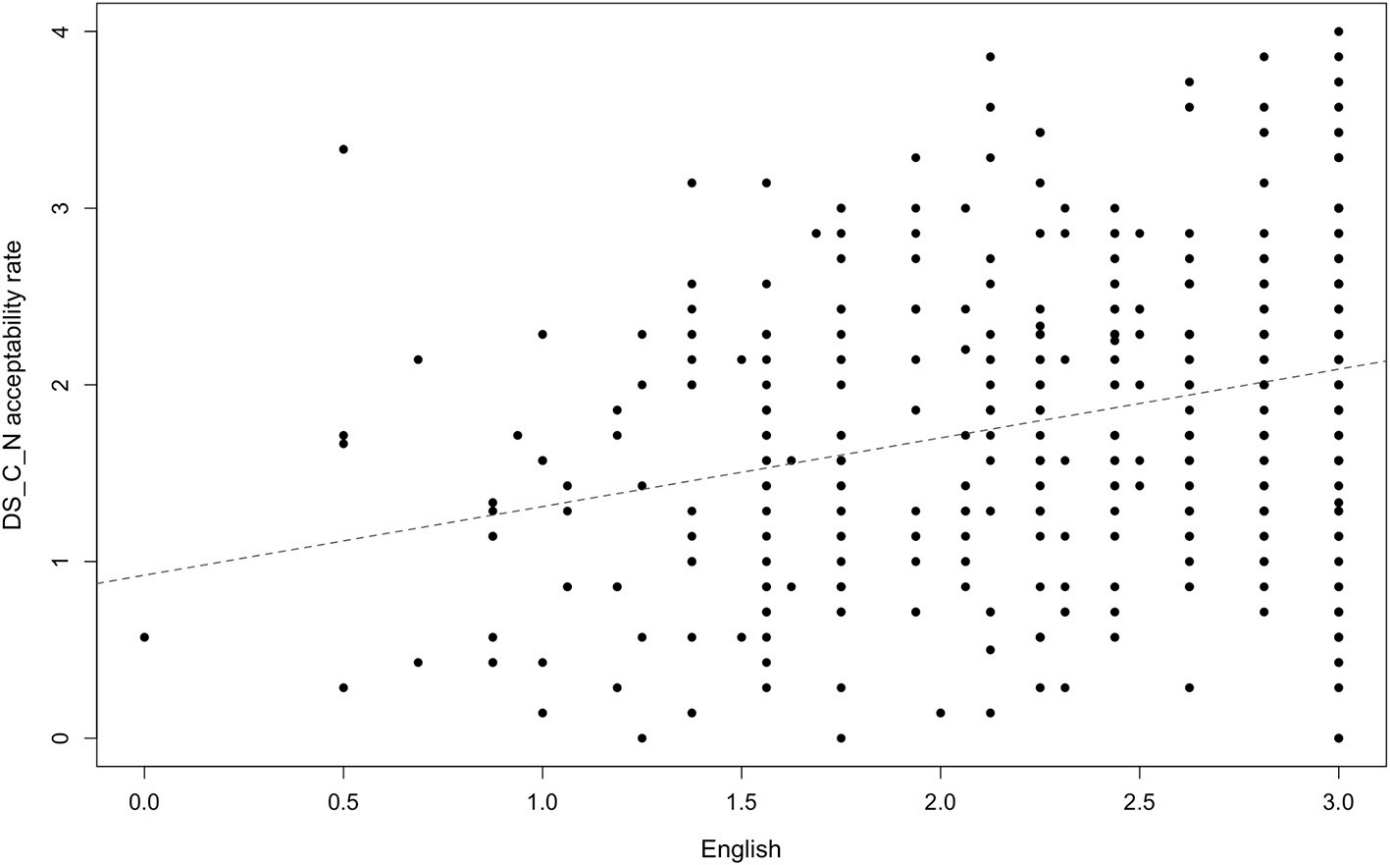
Acceptability rate of *basic*-type adjectives in the non-agreement condition by exposure to and proficiency in English.

**Figure 4:**
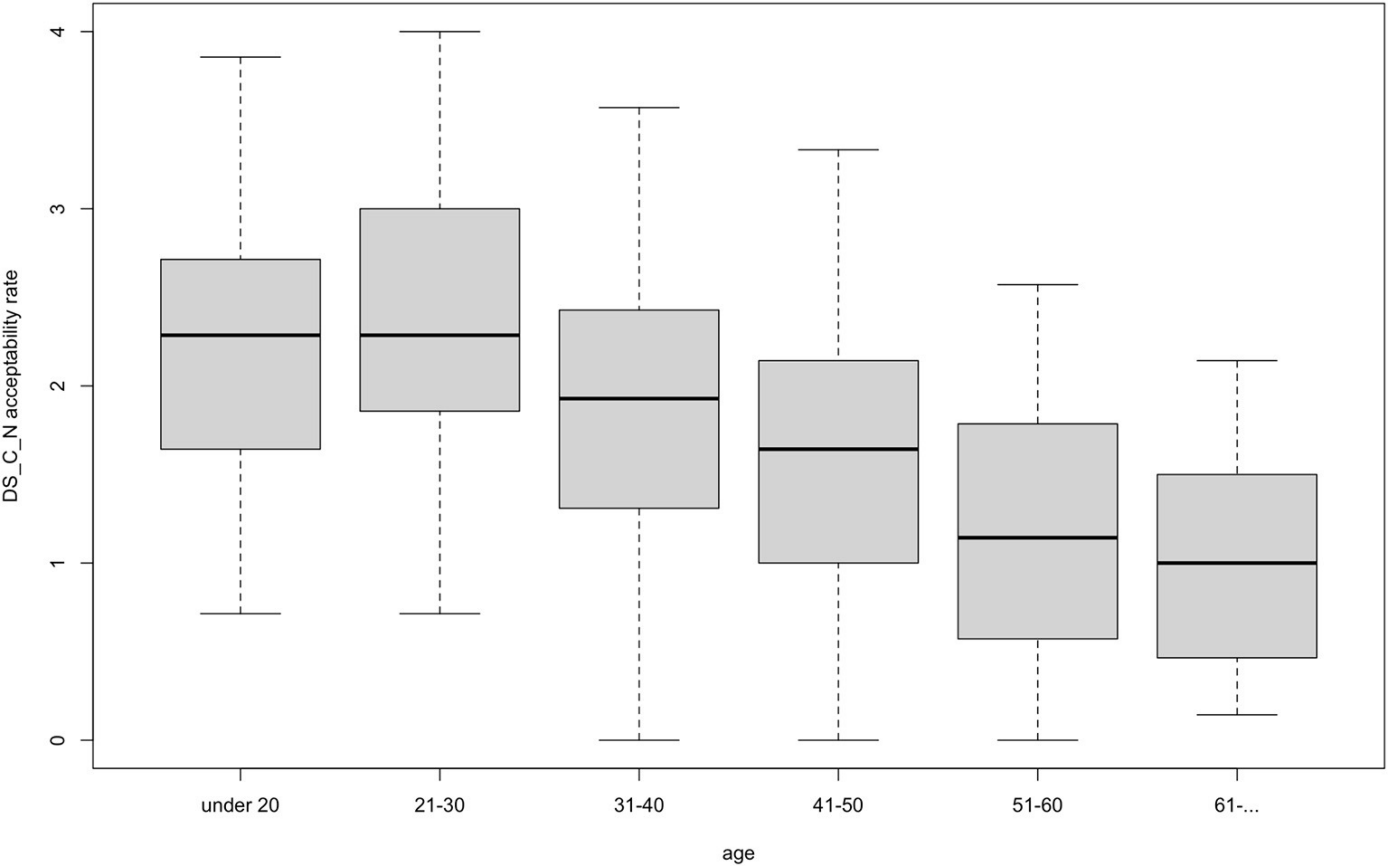
Acceptability rate of *basic*-type adjectives in the non-agreement condition by age.

Our data yielded a very consistent and statistically significant acceptability pattern: all test-sentences were more likely to be accepted by participants who were younger and who had a higher proficiency in and exposure to English. Figure 3 demonstrates this pattern with a reference to one specific adjective type: disyllabic adjectives ending with [i] (*fancy*) in the agreement condition. In the study by Kask (2019) this was the type that always agreed with the noun in number and case, however, among our participants its perception exhibited variation. As “Category and Acceptability Rates” and “Adjective Type and Acceptability Rates” sections demonstrate, the acceptability rate is higher among participants with higher proficiency in and exposure to English. For example, higher levels of English significantly predicted higher acceptability rates for non-agreeing disyllabic adjectives ending with a consonant (*basic*), *b* = 0.39, *t*_(398)_ = 6.08, *p* < 0.001, R-squared = 0.09 (Figure 3). Younger age significantly predicted higher acceptability rates for that type of adjective, *b* = −0.32, *t*_(399)_ = 10.42, *p* < 0.001, R-squared = 0.21 (Figure 4). Across the data, this standard pattern was true for all types in both the agreement and the non-agreement conditions, except for one adjective type, to be discussed below in this section.

**Figure 5:**
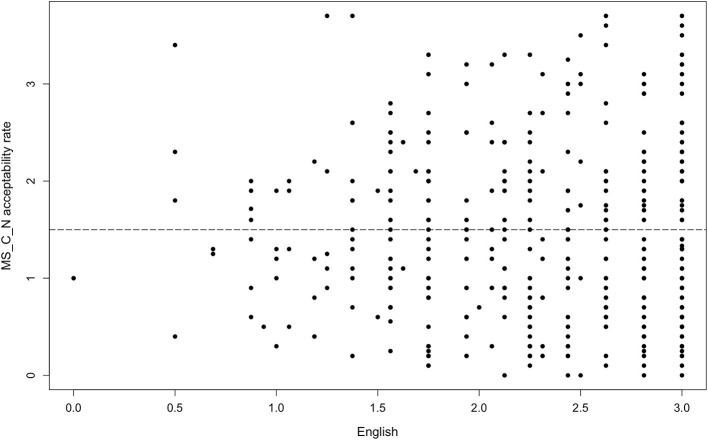
Acceptability rate of *deep*-type in the non-agreement condition by proficiency in English.

**Figure 6:**
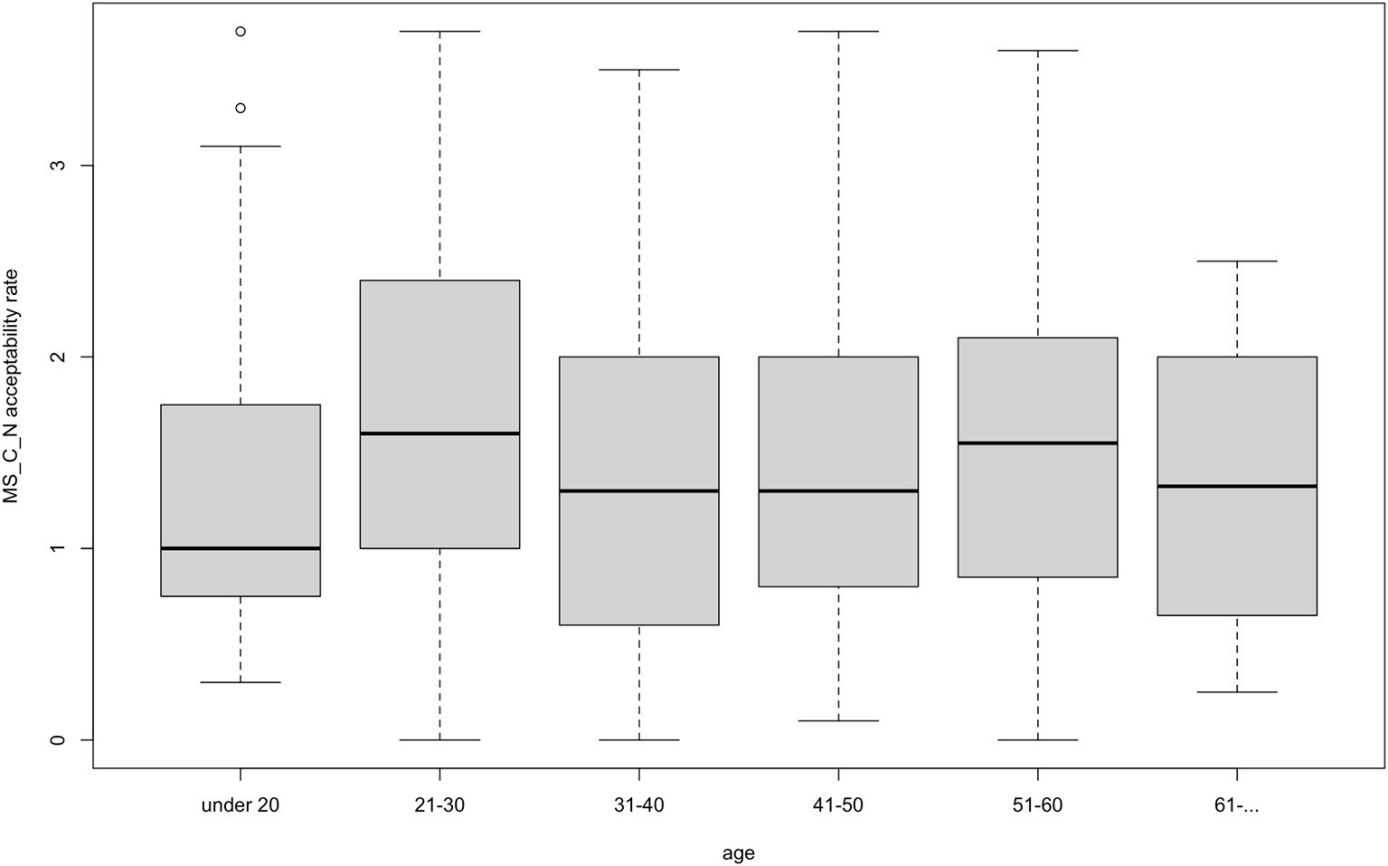
Acceptability rate of *deep*-type in the non-agreement condition by age.

On the other hand, there was one adjective type that did not follow that or any other pattern. Monosyllabic adjectives ending with a consonant (*deep*) did not significantly interact with any factor we tested for in either the agreement or the non-agreement condition. Figures 5 and 6 illustrate the results in the non-agreement condition. Such results contradict expectations also because the adjective *deep* is a conventionalized borrowing in Estonian (spelled *diip*) and is included in the prescriptive Estonian dictionary (Eesti Õigekeelsussõnaraamat ÕS, 2018), which suggests that the adjective would take on Estonian inflectional morphology in the same manner as non-borrowed adjectives.

## Discussion

We looked at the perception of (non-)agreement in bilingual adjective phrases and expected that perception of agreement/non-agreement and real/constructed examples differs among “native speakers” of Estonian. We assumed that acceptability of both agreeing and not agreeing adjectives will be higher in respondents with a high proficiency in/exposure to English. Generally speaking, the hypothesis was confirmed; still, there are some additional factors and some odd cases.

### Sociodemographic Basics

Several sociodemographic observations and factors proved to have a significant impact on the answers.

The first observation is the recurrent combination of young age and high proficiency in English. As described in the Introduction and References therein, younger Estonians are more fluent and more exposed to English. They also are more likely to use the language regularly and to produce bilingual speech, such as blogs and vlogs, as discussed in Kask (2019) and other similar studies demonstrating that bilingual users are typically under 30 years of age. Naturally, we cannot exclude puristic orientation or personal language separation ideals among younger individuals; yet, as expected, high proficiency in English and young age of the participants correlate positively with higher acceptability of all categories of adjectives. This correlation, however, does not justify using these factors interchangeably as young age and high exposure to and fluency in English have a similar but not identical effect.

The second recurrent observation is the relationship between experience of living abroad and fluency in English. Most participants with this experience were also fluent in English, but living abroad was not a prerequisite for English proficiency for young Estonians. Language proficiency and residence in an English-speaking country are often connected but these are not the same: one may achieve a high proficiency in English and use the language frequently without leaving Estonia.

Another sociodemographic factor was residency in Tallinn vs. rural areas. Being a resident of Tallinn was associated with a higher acceptability rate of real sentences when compared to residents in the rural areas. Tallinn residents demonstrated significantly higher acceptability ratings for the non-agreeing disyllabic adjectives ending with a consonant (*basic*) than both rural residents and those currently residing abroad. Tallinn residents also had significantly higher acceptability rates than rural residents for the non-agreeing disyllabic adjectives ending with [i] (*fancy*) and for the agreeing monosyllabic adjectives ending with consonant (*deep*). While the pattern was not statistically significant for all adjective types, we conclude that Tallinn residency is generally positively associated with higher acceptability rates when compared to more rural areas.

The birthplace of respondents also had a relationship with acceptability rates of some adjective types: the agreeing disyllabic adjectives ending with a [i] (*fancy*) and the agreeing monosyllabic type ending with consonant (*deep*). For the latter, respondents born in Tallinn or Tartu rated this adjective type higher than respondents born in smaller Estonian towns. We may have not been able to detect other significant patterns due to low counts in some birthplace categories, but the direction is in line with what we saw with residency: respondents in bigger cities tend to have higher acceptability rates for various types of adjectives.

Gender had some effect on perception of real sentences: participants who identified as males showed slightly lower acceptability of real examples. We have no reasons to associate this pattern with structural reasons and instead interpret this as a reflection of social norms that participants may have oriented to as they completed the tests. The real sentences came from women-written and women-oriented blogs and vlogs on fashion and beauty. We consider these sentences gender-sensitive because the male respondents may find some topics less conventional for their gendered identities. The fact that we found no significant differences between gender categories in the constructed sentences suggests that the overall sentence design was well-balanced and we were able to avoid some possible gender-specific effects in acceptability rates. Gender also affected acceptability of the monosyllabic obstruent ending adjectives (*flat*): participants identifying as females had higher acceptability rates for this adjective type both in the agreement and non-agreement conditions. Currently, we are unable to explain it and a larger corpus study would shed more light on this finding.

### Real vs. Constructed Sentences

While the overall experiment design removed some biases, such as gender bias, there was a minor preference for real sentences. The results showed that real sentences were rated slightly higher than constructed sentences: an average real sentence was rated around 1.8/4, an average constructed was rated around 1.5/4. The difference is minor but statistically significant and therefore something that should be considered. The results may be an artifact of lexical frequency, i.e., certain English adjectives are more frequent in bilingual speech and more conventionalized than others. At present, however, there is no way to check this, as the only existing corpus of English-Estonian bilingual communication has been created by the present authors and is about 300,000 words from fashion and beauty blogs and vlogs, so it represents a rather narrow segment of reality. The respondents may be more familiar with the adjectives used in real sentences, which leads to higher acceptability rates. In addition to that, male respondents assessed constructed sentences somewhat higher, which shifted the results for the entire dataset (see Sociodemographic Basics section for discussion on gendered blogs).

### Category of Adjectives and Acceptability Rate

Increased proficiency in and exposure to English were strongly associated with higher acceptability rate of adjectives in all categories: real and constructed sentences as well as agreeing and non-agreeing adjectives. The relationship was the opposite for age: older speakers had a lower acceptability rate whereas younger speakers demonstrated a higher acceptability rate. The pattern is aligned with a general trend discussed in Sociodemographic Basics section: younger Estonians tend to be more fluent in English, and in our experiment both these factors are strongly linked to increased adjective acceptability.

### Adjective Type and Acceptability Rate

We noticed the same robust pattern, dependent on the age and proficiency in/exposure to English, for all types except the *deep*-type (monosyllabic adjectives ending in a consonant) in the non-agreement condition. Neither age nor English as factors were able to account for varying acceptability rates of this specific adjective type. The factor of birthplace and residence, however, had some influence: respondents in or from larger cities had a higher acceptability rate than those from smaller towns.

### Multivariate Analysis

Given the fact that our corpus consisted of data produced by 401 respondents, we had to narrow down the selection of random effects in the multivariate analysis. We selected our fixed factors based on predictors identified in the bivariate tests (age and English) and added two best random factors (residence and gender). The results show that both age and proficiency are statistically significant predictors of the outcome, with opposite effect: exposure to and proficiency in English positively affect the acceptability outcomes, whereas age slightly decreases acceptability even in respondents with advanced levels of English. These findings corroborate our bivariate tests and confirm our main hypothesis regarding age and English.

### What Cannot Be Explained

Currently, we are unable to explain the exceptional behavior of the above-mentioned *deep*-type. Only the birthplace and residency factors had some impact on its acceptability in the non-agreement condition. Other than that, the acceptability did not depend either on the categories (agreement/non-agreement, real/constructed) or on sociodemographic factors.

### Conclusions

Thus, our research questions are answered in the following way. The first research question, whether there is a difference in grammaticality judgement depending on proficiency in and exposure to English, is answered positively. Proficiency in and exposure to English correlated with higher acceptability of all sentences. Our hypothesis was expanded to include age because proficiency in English is higher among younger speakers.

The answer to the second research question, whether there is a difference in perception of real and constructed examples, is that the difference is only minor. Acceptability of real sentences was somewhat lower among male respondents; this has probably to do with the topic of blogs and vlogs from where the real sentences were retrieved rather than with structural factors. At the same time, overall acceptability of real sentences was slightly higher. A possible explanation may be higher frequency of some adjectives that appeared in the real sentences; at the moment we have no possibility to check this but this is something to be considered in future. In general, the idea to propose both real and constructed sentences to participants as suggested by Verschik (2006) appears reasonable: grammaticality judgement tasks should contain some real examples, otherwise we just obtain a picture of the respondents' opinions of what is possible but cannot relate it to the real speech.

The third research question was, whether there is a difference in perception of the group of English adjectives that fit into Estonian declension system but do not have Estonian inflections in our corpus. We do not have a clear straightforward answer here, as we were not able to identify any clear patterns. In fact, some were the opposite of what one would predict based on compatibility with Estonian declension system, like *deep*, so, apparently structural factors have a limited effect and we should consider other factors, like sociodemographics, and possibly personal preferences.

In addition, multilingual cognition (multicompetence) as well as individual preferences may also play a role. Multilinguals have more linguistic resources and more developed metalinguistic awareness than a monolingual do, and multilinguals can combine elements of both grammars. Hence, a multilingual may not need the same kind of mechanisms of morphosyntactic integration, as was noted by Leisiö (2001). Earlier studies (Zabrodskaja, 2009; Zabrodskaja and Verschik, 2014) demonstrated that multilinguals do not always need to add inflectional morphology of the base language for understanding. Addition of inflectional morphology may be a matter of personal preference and depend on individual proclivity.

The results suggest that “native speakers” do not constitute a homogenous group. From the point of view of sociolinguistics, this is nothing new because different social backgrounds, social networks and linguistic environments affect language use. Still, individuals should not be treated as typical representatives of a socio-demographic group, and their linguistic behavior and metalinguistic awareness may differ. Also from the point of view of contact linguistics, changes in L1 may be perceived differently by bi- and monolingual speakers (Dogruöz and Backus, 2009). Based on the results yielded in our experiment, we believe that even bilingual speakers may differ in their grammaticality judgment. In more general terms, this means that everyone is a “native speaker” of their own idiolect, and because these idiolects have a lot in common, an illusion of a clearly definable ideal native speaker appears.

We identified several directions that would open up avenues for future research aimed at uncovering additional patterns in bilingual constructions. A larger corpus study would allow for a more detailed analysis of the frequency impact on acceptability rates. Another recommendation for an experimental design would be to look more closely into adjective constructions the acceptability ratings of which showed no correlation with the factors tested in this experiment. Finally, while this study focused on English adjectives in Estonian, it would be useful to explore other emergent bilingual constructions.

## Data Availability Statement

The datasets presented in this article are not readily available because de-identified datasets available by request. Requests to access the datasets should be directed to Anna Verschik, annave@tlu.ee.

## Ethics Statement

Ethical review and approval was not required for the study on human participants in accordance with the local legislation and institutional requirements. Written informed consent for participation was not required for this study in accordance with the national legislation and the institutional requirements.

## Author Contributions

The first observation of differential behavior of English adjectives in bilingual phrases was made by AV. Based on this, in 2019, HK conducted an empirical research, demonstrating that this was indeed the case. She identified adjective types and tendencies for (non-)agreement for each type in her data. The idea for the current study was outlined by both HK and AV, based on the method used in an earlier research by AV. The experiment was designed by all three authors. DB oversaw coding work, conducted statistical analyses, and interpreted the results. All authors contributed to the article and approved the submitted version.

## Funding

The research was funded by the Ministry of Education and Research of the Republic of Estonia, Grant EKKD33 Data and corpora of Estonian children and youth multilingual communication.

## Conflict of Interest

The authors declare that the research was conducted in the absence of any commercial or financial relationships that could be construed as a potential conflict of interest.

## Publisher's Note

All claims expressed in this article are solely those of the authors and do not necessarily represent those of their affiliated organizations, or those of the publisher, the editors and the reviewers. Any product that may be evaluated in this article, or claim that may be made by its manufacturer, is not guaranteed or endorsed by the publisher.

## Footnotes

^1^There are various corpora of Estonian, for instance, of periodicals and fiction and of colloquial Estonian (closed to general public). The former contain edited text (from the point of view of a monolingual norm), the latter comprise phone conversations. In either of them, synchronic contact-induced language change phenomena are not annotated. The compilers of the corpora and researchers working with them are not interested in contact linguistics and multilingualism research. Estonian linguistics research community is tiny and there are less than a dozen researchers who work in the field of contact linguistics, so our own corpus is the only one of a kind.
