# Supplementary material for: English Adjectives and Estonian Nouns: Looking for Agreement?
Source: Front Psychol. 2021 Oct 14;12:735232. doi: 10.3389/fpsyg.2021.735232 (PMC8552072; doi:10.3389/fpsyg.2021.735232)

Appendix A: Survey questions (translated from Estonian into English)

1. Your age:
    - under 20
    - 21-30
    - 31-40
    - 41-50
    - 51-60
    - 61-...
2. Birth of place (country, city): open answer
3. Place of residence (country, city): open answer
4. Have you lived in an English-speaking country for more than a year?
    - Yes
    - No
5. Gender
    - Male
    - Female
    - Other
6. Highest educational degree
    - Basic education
    - Secondary education
    - Secondary specialized education
    - Higher education
7. Current employment status
    - High school student
    - College student
    - Maternity/paternity leave
    - Unemployed
    - Public servant or employee
    - Other
8. Proficiency in English
    - No proficiency
    - I understand but cannot speak
    - I understand everything and can communicate to an extent
    - I am fluent
9. How often do you read or hear English?
    - All the time
    - More than couple of times a week
    - Couple of times a week
    - Couple of times a month
    - Rarely
    - Never
10. How often do you speak or write in English?
    - All the time
    - More than couple of times a week
    - Couple of times a week
    - Couple of times a month
    - Rarely
    - Never

Appendix B: Sociodemographic overview of 401 respondents.

Gender	Male	Female	Other			
	61	336	1			
Birthplace	Tallinn	Tartu	Urban	Rural	Abroad	
	109	64	118	103	5	
Residence	Tallinn	Tartu	Urban	Rural	Abroad Now	Abroad Ever
	154	80	29	95	41	49
Age	Under 20	21-30	31-40	41-50	51-60	61-…
	20	77	116	110	52	24

Appendix C: English proficiency overview for 401 respondents.

Fluency	None	Passive	Passive and somewhat active		Fully fluent
	3	25	133		239
Active use	Seldom	Monthly	Weekly	Daily	All the time
	73	77	64	79	99
Passive use	Seldom	Monthly	Weekly	Daily	All the time
	11	11	47	100	228
Normalized score based on self-reported fluency, active, and passive use of English					
Score	0.5	0.685	0.875	0.9375	1
Respondents	5	2	7	1	5
Score	1.0625	1.1875	1.25	1.375	1.5
Respondents	4	5	5	13	2
Score	1.5625	1.625	1.6875	1.75	1.9375
Respondents	24	2	1	32	17
Score	2	2.0625	2.125	2.25	2.3125
Respondents	1	10	21	29	9
Score	2.4375	2.5	2.625	2.8125	3
Respondents	27	7	36	46	89

Appendix D: Test sentence examples for adjectives ending with *-e* in spelling but with consonant in pronunciation in the agreement (A) and the non-agreement (N) conditions.

Type	Agreement (A)	Non-agreement (N)
Ending with vowel in spelling but with consonant in pronunciation (V_C), Estonian prototype *kõrb* 'beige	** Ole positiivselt kade pigem selle peale, kellel on awesome'id sõbrad.*<br>awesome'i-d sõbra-d<br>awesome-PL.NOM friend-PL.NOM<br>'rather be positively jealous of somebody who has awesome friends'	** Ole positiivselt kade pigem selle peale, kellel on awesome sõbrad.*<br>awesome sõbra-d<br>awesome friend-PL.NOM
	Minu favourite'id teksad on kunagi ammu ostetud Mustangist.<br>favourite'i-d teksa-d<br>favorites-PL.NOM jeans-PL.NOM<br>'my favorited jeans are bought from Mustang a long time ago'	*Minu favourite teksad on kunagi ammu ostetud Mustangist.*<br>favourite teksa-d<br>favourite jeans-PL.NOM
	Sellega saab nii awesomeid kaadreid.<br>awesome-i-d kaadre-i-d<br>awesome-PL-PART cadres-PL-PART<br>'this takes awesome pictures'	*Sellega saab nii awesome kaadreid.*<br>awesome kaadre-i-d<br>awesome cadres-PL-PART
	Minu uued nude'id kevadsaapad.<br>nude'i-d kevadsaapa-d<br>nude-NOM.PL spring boot-NOM.PL<br>'my new nude spring boots'	*Minu uued nude kevadsaapad.*<br>nude kevadsaapa-d<br>nude spring boot-NOM.PL
	Loosin välja ühe beige'i nokatsi.<br>beige'i nokatsi<br>beige.GEN cap.GEN<br>'I will give away a beige cap'	*Loosin välja ühe beige nokatsi.*<br>beige nokatsi<br>beige cap.GEN
	Õige pea näete, mis insane'id tegevused mul praegu käsil on.<br>insane'i-d tegevuse-d<br>insane-NOM.PL activity-NOM.PL<br>'soon you will see what kind of insane activities I am on to'	*Õige pea näete, mis insane tegevused mul praegu käsil on.*<br>insane tegevuse-d<br>insane activity-NOM.PL
	Need on väga simple'id ja samas odavad tooted, minu nahale ideaalsed.<br>simple'i-d toote-d<br>simple-NOM.PL product-NOM.PL<br>'these are very simple and at the same time cheap products, perfect for my skin'	*Need on väga simple ja samas odavad tooted, minu nahale ideaalsed.*<br>simple toote-d<br>simple product-NOM.PL
	Need cute'id saapad skoorisin kaltsukast 3 euroga.<br>cute'i-d saapa-d<br>cute-NOM.PL boot-NOM.PL<br>'I got these cute boots from a secondhand store for 5 euros'	*Need cute saapad skoorisin kaltsukast 3 euroga.*<br>cute saapa-d<br>cute boot-NOM.PL

Real examples (R) are preceded by an asterisk (*).

FIGURE 7| Self-reported English proficiency / use (calculated based on data from Appendix C).

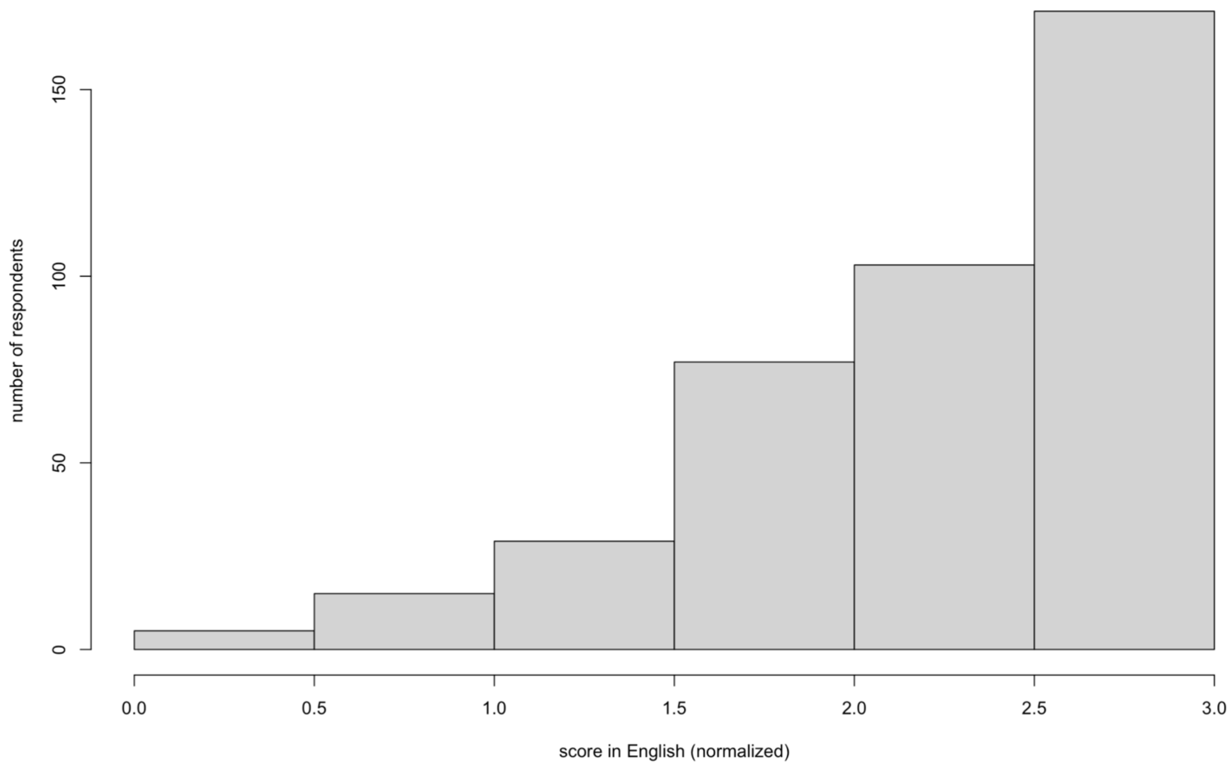

Supplement: Supplementary file 1 [file Data_Sheet_1.pdf]
